# Effectiveness of mobile technology and utilization of maternal and neonatal healthcare in low and middle-income countries (LMICs): a systematic review

**DOI:** 10.1186/s12905-023-02825-y

**Published:** 2023-12-11

**Authors:** Prasenjit De, Manas Ranjan Pradhan

**Affiliations:** Department of Fertility and Social Demography, International Institute for Population Sciences (IIPS), Govandi Station Road, Deonar, Mumbai, 400088 Maharashtra India

**Keywords:** Mobile Health, Mobile phone, Maternal health, Neonatal health, LMICs

## Abstract

**Background:**

Maternal and neonatal mortality are important indicators of the development of a nation and pose a severe health concern, especially in developing and Low and Middle-Income Countries (LMICs). Healthcare providers use various mobile technologies as tools to provide antenatal, delivery, and postnatal care and thereby promote maternal and child health. We conducted a systematic review to critically assess the existing literature on the effectiveness of mobile phone technology in maternal and neonatal healthcare (MNH) utilization, especially in LMICs in Asia and Africa.

**Methods:**

A systematic search strategy was developed, and Boolean combinations of relevant keywords were utilized to search relevant literature on three electronic databases (PubMed/Medline, Scopus, and Google Scholar) from 2012 to 2022. After assessing the inclusion and exclusion criteria, 25 articles were selected for systematic review. A narrative synthesis strategy was applied to summarise the information from the included literature.

**Results:**

This review reveals that research and evaluation studies on mobile phone or Mobile Health (mHealth) and MNH service utilization substantially varied by research designs and methodology. Most studies found that mobile phone technology is highly appreciable in improving several MNH indicators, especially in LMICs. Despite the identified benefits of mobile technology in MNH utilization, some studies also mentioned challenges related to technology use and misuse, rich-poor discrimination, and disparity in phone ownership need to be addressed.

**Conclusion:**

There is constantly increasing evidence of mobile counseling and the use of digital technology in the MNH care system. Public health practitioners and policymakers need to make efforts to smooth the functioning of technology-based healthcare services, considering all the issues related to the confidentiality and safety of health-related data on the Internet.

**Supplementary Information:**

The online version contains supplementary material available at 10.1186/s12905-023-02825-y.

## Introduction

Reducing maternal and neonatal mortality is a significant public health challenge in the 21st century. Maternal mortality is an essential indicator of the development of a nation [1] and poses a severe health concern, especially in developing and Low and Middle-Income Countries (LMICs). Most (94%) of all maternal deaths occur in LMICs [2]. Improving newborn and child health also remains a top-level priority for LMICs. Two regions, Sub-Saharan Africa and Southern Asia, account for more than 80% of the 5.0 million under-five deaths in 2020 [3]. The Sustainable Development Goals (SDGs) aim for a reduction of the maternal mortality ratio (MMR) to below 70 per 100,000 live births and infant mortality by as low as 12 per 1,000 live births by 2030 [4]. The use of mobile technology in Maternal and Neonatal Healthcare (MNH) is one of the several attempts introduced in many LMICs to meet the SDG target of reducing maternal and neonatal deaths.

Mobile Health (mHealth), i.e., using wireless, portable Information and Communication Technologies (ICTs) to support health and health care [5], is widely promoted as a tools to decrease maternal and child mortality [6]. Because of the pervasiveness and ease of mobile communication, mobile applications are used to manage diseases and promote healthy behaviors [6]. In recent years, the use of mobile technology in LMICs has expanded fast [7], and this increased access has the potential to improve healthcare delivery [8, 9]. The availability of mobile phones and the usage of mHealth have been expected as a potential remedy to many of the problems developing countries are currently experiencing in their healthcare delivery systems [10]. mHealth has the potential to reduce inequalities in care, with the overarching goal of improving access to and quality of obstetric care through a variety of applications that aim to facilitate communication between clients and providers, promote women’s behavioral change, simplify and extend training, and assist in data collection [11].

Using smartphones and mobile-based applications opens a new horizon in healthcare management systems, including diagnosis, risk reduction, health education, self-care, and home treatment, improving health outcomes in patients with chronic diseases [12]. In recent years, especially in developing countries, cost-effective delivery of self-management services through mobile technology has emerged as a novel approach for people living with Human Immune-deficiency Virus (HIV) [13], such as appointment and medication reminders [14]. Furthermore, it has been found that mobile-based follow-ups improved trust in treatment and enhanced performance in HIV patients [15]. Healthcare providers use various mHealth technologies as tools to promote maternal and child health. These tools are used in communities to provide antenatal, delivery, and postnatal care [6]. A mobile-based consultation service, which can overcome many barriers to accessing appropriate health care, could be utilized as a health promotion tool to raise awareness among women and their families in remote settings [16, 17].

A plethora of research has been published exploring the use of mobile technology in healthcare delivery, but it differs from the scope of this review. There is evidence of research that focuses on developed countries related to maternal health and mobile technology, but only a few are based on developing countries and LMICs. A review published in 2016 tried to synthesize knowledge about the effectiveness of mHealth intervention only in Lower-Income countries to address maternal health [11]. Other studies focus on interventions related to community health workers [18, 19]. Several studies have attempted to map the state of the evidence relevant to mHealth for maternity, neonatal, and child health (MNCH) in LMICs. Still, to the best of our knowledge, no rigorous systematic review exists on the effectiveness of mobile technology and utilization of MNH in LMICs, incorporating experimental and non-experimental studies. The need for this review is determined after confirming that the most recent literature from other systematic reviews has been taken on a vast scope while attempting to establish a relationship between mobile phone use and MNH, particularly in developing countries and LMICs [17, 19]. The present systematic review builds on these efforts more rigorously and comprehensively by including a clear systematic search strategy and capturing a comprehensible picture of the state of the evidence for the role of mobile phones in MNH care utilization. Specifically, this systematic review aims to (a) critically assess the existing literature on the effectiveness of mobile phone technology in MNH care utilization and (b) synthesize the existing evidence on mobile phone and MNH utilization, especially in LMICs.

## Methodology

The methodological section for this study includes study design, a well-defined search strategy, electronic database criteria for selecting research papers, data extraction, synthesis, and a complete diagram of the selection process using Preferred Reporting Items for Systematic Reviews and Meta-Analyses (PRISMA) guidelines.

### Study design

The present study incorporates a systematic review of the chosen papers and a narrative synthesis technique to discover common themes and summarize the results.

### Search strategy and keywords

A systematic search strategy was developed to identify relevant literature to fulfill this study’s objective. Three electronic databases, e.g., PubMed/Medline, Scopus, and Google Scholar, were searched for articles published between 2012 and 2022 using the following keywords.

A. “Mobile phone” OR “Mobile technology” OR “Smartphone” OR “Mobile Health” OR “mHealth”
B. “Maternal health” OR “maternal health services” “Maternal health service utilization”
C. “Neonatal health” OR “Neonatal health services” OR “Neonatal health service utilization”
D. “Developing countries” OR “Low-and middle-income countries” OR “LMICs”
E. [A] AND [B]
F. [A] AND [C]
G. [A] AND [B] AND [D]
H. [A] AND [C] AND [D]
I. [A] AND [B] AND [C] AND [D]

We initially identified a total of 1526 records through a database search.

### Study selection

**Fig. 1:**
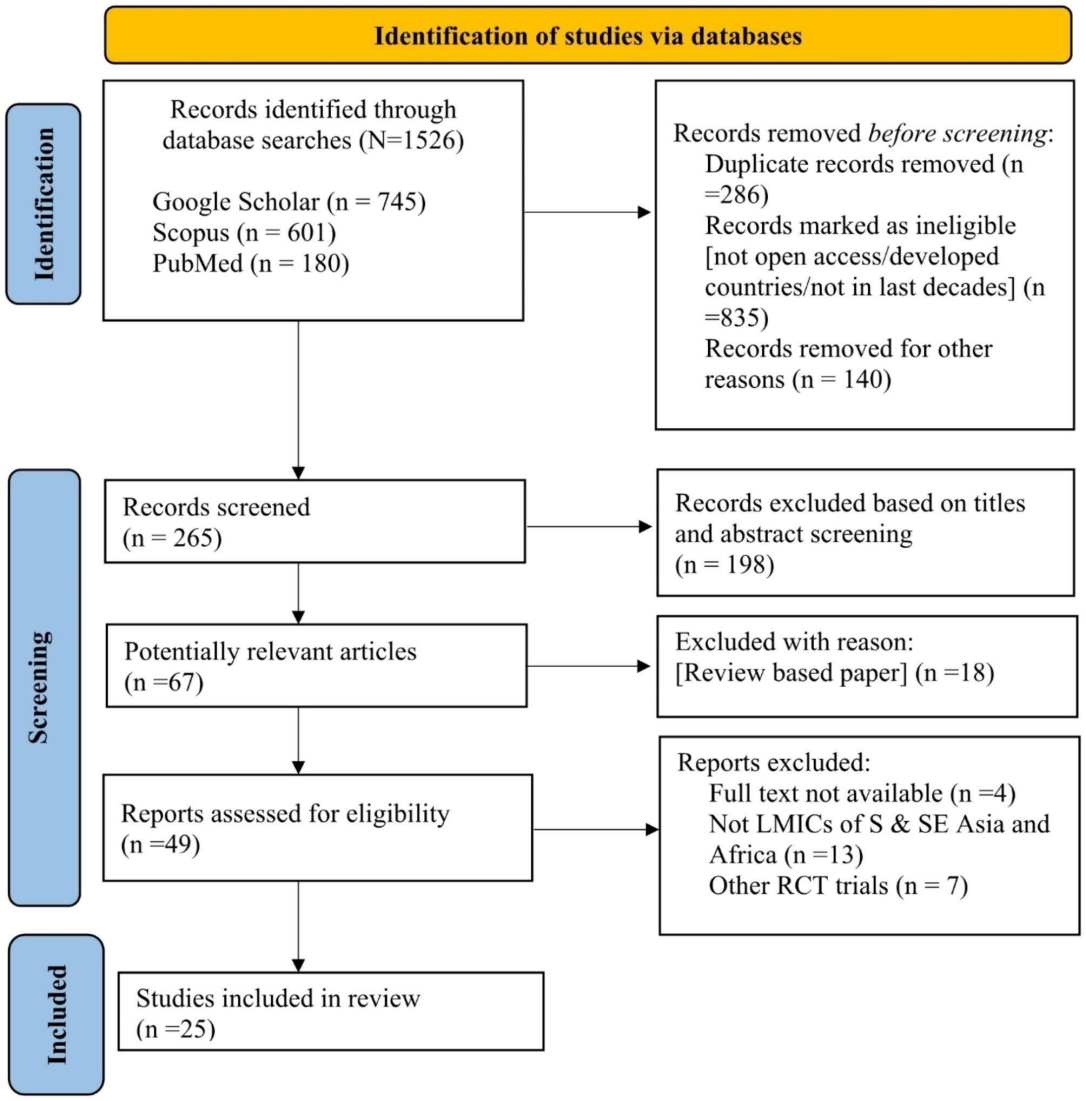
PRISMA diagram illustrating the study selection process

In this systematic review, articles were incorporated if the title and abstract suggested the results of original research studies using a quantitative, qualitative, or mixed-method design. Reports, commentaries, review articles, and letters were excluded. Both authors did the abstracts screening of all articles retrieved from the search. The inclusion and exclusion criteria for selecting papers are given in Table 1. We included only full texts of potentially relevant articles published in English. The search for articles persisted until duplicate articles were identified. After assessing the initial criteria, 265 records were screened, among which 198 were excluded based on a clear title and abstract screening. Then, 67 potentially relevant articles were identified. Among them, 42 articles were removed with valid reasons following the PRISMA guidelines (as shown in Fig. 1). Finally, 25 articles were included for review. We considered cross-sectional, cohort, case-control, and experimental studies, including randomized controlled trials. MENDELEY (V 1.19.8) was utilized to organize and store the literature.

**Table 1:** Inclusion and Exclusion Criteria

	Inclusion criteria	Exclusion Criteria
Topic	Papers identifying the relationship between mobile and MNH care utilization Papers evaluating mobile phone intervention addressing maternal health outcomes Impact assessment studies aimed at the skill development of healthcare providers related to MNH service	Papers addressing other mHealth or e-health interventions (e.g., telemedicine, telecommunication, etc.) Papers focused on other health outcomes not related to MNH.
Study setting	Developing and low and middle-income countries (LMICs) Focusing only on Asian and African countries.	Developed or high-income countries Countries located other than Asia and Africa.
Publication types	Paper published in peer-reviewed journals	Policy briefs, commentaries, training workshops, and special or technical reports.
Access	Open access Full text available	Not open access No full text is available
Language	Only English	Other than English
Types of study	Cross-sectional, cohort, case-control, experimental, and randomized control trials.	Review based papers
Publication date	Between 2012 to 2022 (September)	Before 2012 and after September 2022

### Data extraction

After the selection procedure, data extraction was done in the form of a table to summarize the results of selected studies. The authors extracted the data relating to the authors of the published paper, year of publication, primary objective of the study, study participants, name of the application, study design and methodology, major findings, and critical remarks. The relevant data was recorded in a pre-formatted data collection form. Meta-analysis was not considered appropriate for this review because of the wide diversity of studies concerning study settings, study aim, research design, study population, types of interventions, and outcomes (supplementary file 1).

### Risk of bias assessment

Nevertheless, potential selection, publication, and language biases were considered while searching for articles. As part of the data extraction process, we evaluated the study quality by assessing the alignment of the study purpose to the study design and methods and the evaluation of methodological soundness. The selected articles’ quality was acceptable, with appropriate methods to address articulated research aims and questions.

### Synthesis

After extracting data, a narrative synthesis strategy was performed to synthesize the findings of selected studies. Due to the diverse range of studies in this systematic review, it was decided that a narrative synthesis strategy constitutes the most suitable method for summarizing the findings. Narrative synthesis has been recommended for reviews encompassing results from various heterogeneous studies when statistical meta-analysis and meta-ethnography alone are not feasible [20]. The method entails an initial synthesis through the thematic analysis involving searching of studies, listing, tabulating, and presenting results in tabular form. Then, the results were discussed again and structured into themes. The first author conducted initial synthesis by thematic analysis, with further analytical input from the second author. Differences were resolved by consensus. The summarized results of the present study were discussed according to the emerging themes in the result section.

## Results

### Profile of the studies included in the review

All studies were based on LMICs of either Asia or Africa. Out of 25 included pieces of literature, nine were undertaken in India [7, 21–28], three in both Bangladesh [16, 29, 30] and Nigeria [6, 10, 31], and one each from Afghanistan [32], Congo [33], Madagascar [34], Malawi [35], Pakistan [36], Timor-Leste [37], and Uganda [38]. The other three studies were multinational, including countries from Asia [39], Africa [40], or both [41].

This review reveals that research and evaluation studies on a mobile phone or mHealth and MNH service utilization substantially varied in research designs and methodology. Most were quantitative than qualitative [24, 38], while three studies [16, 23, 42] incorporated both methods.

Thirteen studies were non-experimental, typically following a cross-sectional design to describe or identify any relationship between mobile phone ownership and maternal health and mobile phone-related MNH service utilization. Three studies were experimental, designed with randomization of control and intervention groups [7, 26, 27]. In comparison, five studies were quasi-experimental, designed with a control group and a traditional paper-based control intervention without randomization of participants.

### Access to and use of mobile phones and maternal healthcare utilization

Some of the selected cross-sectional studies, based either on small-scale surveys or Demographic and Health Survey (DHS) data, explore the relationship between access to mobile phones and maternal healthcare utilization in terms of antenatal care (ANC), postnatal care (PNC), vaccination, institutional birth, skill birth attendance, and other related healthcare service utilization. Women who have access to mobile phones [31, 37, 41] or use them for health-related purposes [29] and who reported receiving family planning messages through the Internet and mobile phone [35] had higher odds of having timely and adequate ANC care, institutional delivery [29, 35, 37], skilled attendance at birth [31, 37, 41], immunization during pregnancy [41], and timely postnatal care [22, 37, 41]. Most of the study findings were the same across the study settings of LMICs, thereby proving the validity of such conclusions.

However, some studies mentioned that after adjusting for socioeconomic variables, mobile phone ownership or access was not significantly associated with adequate ANC, skilled birth attendance [37], and postnatal check-ups [31, 37], while another study conducted in India claimed that mobile phone access was associated with PNC only in urban settings [22]. Moreover, a study in sub-Saharan Africa revealed that access to maternal healthcare was not affected by mobile phone ownership, yet it was a relevant mediator of contraceptive use [40].

### Newborn child or neonatal healthcare and mobile phone

Newborn child or neonatal healthcare is also closely associated with maternal healthcare, reciprocally known as MNH services. A few reviewed studies found that women who had access to a mobile phone or used a mobile for health-related purposes had significantly higher odds of receiving neonatal check-ups [30, 41] and child immunization [41]. Despite those positive relations, some studies found that phone ownership was not significantly associated with newborn check-ups [37], breastfeeding, and care-seeking for acute respiratory infections or diarrhea in children under five [41].

### Specific mHealth intervention and MNH utilization

With the fast spread of mobile health technologies, there is a growing interest in determining how mobile technologies can successfully improve maternal health outcomes, specifically maternal health-seeking knowledge and behaviors [23]. For this purpose, several mHealth projects have already been implemented in LMICs.

### Improving MNH service utilization and access to information

Mobile health or mHealth projects are implemented to increase MNH service utilization, thereby reducing maternal mortality in developing countries and LMICs. Additionally, few programs are designed to empower women by increasing access to health-related information. A study in Nigeria found that compared to the control group, women who got the mobile health intervention had a higher likelihood of using postnatal care visits [10]. Another qualitative study in Uganda reported that intervention was acceptable as it helped participants adopt excellent maternal health practices, provided clinic appointment reminders, and facilitated communication with healthcare providers [38].

Exposure to voice messages or Short Message Service (SMS)-based services improved access to and use of recommended services for high-risk pregnancies [27], thereby improving maternal health outcomes [34]. Another study found mobile text messaging services aimed at increasing knowledge about MCH are very effective and significantly increased in the knowledge related to maternal health services [26]. A mHealth intervention that provided consultation services for maternal, neonatal, and infant care in Bangladesh revealed improvement in people’s decision-making during emergencies and reduced delays in reaching health facilities [16]. Another intervention aimed to provide MNH-related information to a husband’s mobile phone implemented in Uttar Pradesh, India, found that if husbands discussed the messages with family members, the odds of wives’ receiving ANC, PNC, and delayed bathing of newborns improved significantly [28].

### Community health workers (CHWs) and mHealth

Various projects introduced mobile phones to improve the capacity of the community and frontline health workers to improve the coverage, coordination, and quality of MNH care services. Community health workers reported that the particular intervention helped them work better, increase their confidence [23], and improve their community status [24]. A project titled ReMinD, implemented in Uttar Pradesh, increases health workers’ capacity to deliver quality counseling to pregnant women and found a significant increase in self-reporting of complications during pregnancy and after delivery [25]. An internet-enabled phone strongly predicted higher mHealth knowledge and a positive attitude toward using mHealth for providing MNH service among primary healthcare providers [6]. An intervention implemented in Bihar, India, found that the specific intervention increased the number of frontline health workers’ home visits during the third trimester [7].

### Challenges related to Mobile technology and MNH utilization

Despite the identified benefits of mobile technology in MNH utilization, some challenges related to technology use, misuse, and disparity of phone ownership still need to be addressed. Two studies found that women from a higher economic background were more likely to use Internet-based smartphones for healthcare-seeking purposes than their counterparts [30, 39], conforming to the prevailing rich-poor discrimination. A study in India found that there were sociocultural and technological barriers and mistrust in implementing a mHealth project [24]. Providing educational videos/audios, appointment reminders, and a calling function was insufficient if there was no money to purchase nutritional supplements needed by pregnant/breastfeeding women, unavailability of adequate transport facilities during emergency care, and out-of-pocket expenditure of some medical treatment [38]. Other limitations were related to phone sharing, where the fact that a message was delivered did not imply that the woman included in the study read it and that her behavior was influenced by the SMS [38].

## Discussion

This systematic review examines mobile phone access to and use in MNH care, particularly in LMICs, using a thorough literature search technique and reveals a positive trend toward increased use of experimental design, especially in the past few years. An earlier review explored this topic but differed from the present study’s scope, inclusion, and exclusion criteria [11]. Despite some overlap, this review incorporates research evidence on mobile phone use in MNH care utilization by including experimental and cross-sectional studies. Findings from the cross-sectional studies have been discussed to capture the current status of the accessibility of mobile phones and the relationships between MNH care utilization and mobile phones. Large sample sizes of the cross-sectional designs help to generalize the findings across the study settings. Most studies demonstrated that women with access to mobile phones are more likely to receive ANC, PNC, institutional delivery, and immunization during pregnancy. On the other hand, experimental studies help to summarize the effectiveness of mobile technology in delivering MNH care. The combined results of the experimental studies show that mobile technology significantly improved MNH care services among the intervention groups and helped CHWs provide quality care. However, special attention is needed to the risk of potential biases in experimental studies, such as small sample size and target-based implementation. Although findings from both study designs align with the effectiveness of mobile technology in delivering MNH care, only a few experimental studies mentioned the challenges in using mobile technology to provide adequate healthcare services, such as economic and technological barriers and mistrust among the participants and thereby differ from the findings of other experimental studies.

Most studies found that using mobile phone technology is highly appreciable to improve several MNH indicators, including timely and adequate numbers of ANC and PNC visits, institutional delivery, vaccination during pregnancy, newborn immunization, and newborn check-ups. This review’s findings also agree with previous studies [11, 43] on effectively utilizing digital technology and mobile phones to address maternal health, especially in LMICs. Although the effectiveness of mHealth is well-documented, few studies have attempted to show how frequently mothers or pregnant women use phones to access MNH services, what problems they encounter, and how satisfied they are with mobile-based MNH care-seeking without intervention.

The rapid global adoption of mobile phones could reduce MNH service utilization delays. It also enables communication for medical purposes in isolated areas. According to the available literature, there is considerable opportunity to combine traditional and biomedical health care and connect different levels within a health care system, provided that women are not constrained owing to their social status, lack of resources, or transportation [43]. Using mobile phones, partnerships between governments, technologists, non-governmental organizations, academia, and industry can improve healthcare delivery in LMICs [44]. Mobile technology also shows excellent potential to enhance CHW’s knowledge and practices to provide MNH care to the beneficiaries. This review also reveals that CHWs increasingly use mHealth tools to improve MNH service delivery, which was also reported by an earlier study mainly focused on CHWs [45].

Many previous studies found that the use of smartphones or the Internet is an effective tool for communicating with patients [44], sending medication reminders to patients [14], and enhancing their performance [15]. Mobile technology also enhances self-care for patients with chronic diseases [12], thereby supporting public health services [13]. Some other studies argued for a clear-cut policy related to social media use in patient care to ensure the utmost safety of the patient [46]. However, this review found a need for studies prioritizing patients’ safety while accessing healthcare using the Internet or mobile phone. Some studies are also silent on adverse outcomes that intervention related to mHealth may have caused, which is useful to know the actual effectiveness of such a program. This review attempted to include both quantitative and qualitative studies related to the particular topic. However, very few are based on qualitative design to support quantitative findings, and these studies do not explore the investigation’s qualitative perspective. This finding is consistent with another review focusing on the effectiveness of mHealth intervention among CHWs [45].

### Limitations and strengths

There are several limitations of this systematic review. This review restricts itself to studies published in English, concentrating only on open-access articles, and only PubMed/Medline, Scopus, and Google Scholar databases were used to search for appropriate studies. Due to this fact, there is a possibility of missing out on relevant studies on other databases and non-English language articles. Similar concerns are mentioned in a past systematic review [47]. The selection of only 25 articles may only represent part of the scenario of the existing body of literature on this particular issue. Due to the heterogeneity of methods, units of analysis, and designs in this continuously evolving field of research, a meta-analysis is not feasible. Despite these limitations, this review has several strengths. It provides rigorous and comprehensive knowledge related to the effectiveness of mobile technology in MNH care utilization, especially in LMICs. It has successfully captured the experimental and non-experimental design-based studies on this topic through a systematic way of analysis. It also incorporates a diverse perspective like MNH care-seeking and its relation to mobile technology, a view from CHWs to know the actual scenario.

## Conclusion and recommendations

This review suggests the potentiality of mobile phone-based technology use and uptake of several MNH care utilization in LMICs. The overall evidence of the included literature is weak, and the results are sometimes inconsistent to draw a robust conclusion on the extent to which mobile phones are feasible for maternal health outcomes. However, it has been clear that mobile technology is widely used by service seekers and providers to improve MNH care utilization, especially in resource-constrained settings. The rapid use of mobile phones and their usage in healthcare created several opportunities for remote care. Based on the evidence and gaps identified through this review, future studies need to consider and incorporate questions like how an individual uses mobile or smartphones to access MNH services and how this technology-based healthcare service affects the individual. Moreover, the knowledge, attitude, and practices of service providers and beneficiaries relating to mobile technology and MNH utilization in a particular setting need to be explored in detail to better understand the situation. In this era of digitalization, technology-based healthcare services become an integral part of our society. So, public health practitioners and policymakers need to make efforts to smooth the functioning of technology-based healthcare services, considering all the issues related to the confidentiality and safety of health-related data on the Internet.

### Electronic supplementary material

Below is the link to the electronic supplementary material.

Supplementary File 1: Summary of Included Studies

## Data Availability

The dataset (s) supporting the conclusions of this article is (are) included in the additional file (s).

## Abbreviations

ANC: Antenatal care
CHWs: Community Health Workers
DHS: Demographic and Health Surveys
ICTs: Information and Communication Technologies
LMICs: Low and Middle-Income Countries
mHealth: Mobile Health
MMR: Maternal Mortality Ratio
MNCH: Maternal, Neonatal, and Child Health
MNH: Maternal and Neonatal Health
PNC: Postnatal care
PRISMA: Preferred Reporting Items for Systematic Reviews and Meta-Analyses
SDGs: Sustainable Development Goals
SMS: Short Message Service
