# Supplementary material for: Effectiveness of mobile technology and utilization of maternal and neonatal healthcare in low and middle-income countries (LMICs): a systematic review
Source: BMC Womens Health. 2023 Dec 11;23:664. doi: 10.1186/s12905-023-02825-y (PMC10714653; doi:10.1186/s12905-023-02825-y)
Supplement: Supplementary file 1 — Summary of Included Studies [file 12905_2023_2825_MOESM1_ESM.docx]

| **Supplementary File 1: Summary of Included Studies** | | | | | | |
| --- | --- | --- | --- | --- | --- | --- |
| **SL. NO.** | **TITLE** | **AUTHOR (S) & YEAR** | **MAJOR OBJECTIVE** | **METHODOLOGY**   - **Study Setting** - **Data Source** - **Study Design** - **Sample Size/Study participants/Main users** - **Analysis Method** | **MAJOR FINDINGS** | **LIMITATION**  **&**  **CRITICAL REMARKS** |
| 1 | Association Between Mass Media Use and Maternal Healthcare Service Utilisation in Malawi | Yanjie Wang et al.,  2021 | To assess the role of exposure to family planning information through various mass media on the utilization of maternal healthcare services | - Malawi country - DHS - Cross-sectional at the individual level - 13217 women (aged 15 to 49 years) - Multivariate logistic regression model | - Women who reported receiving family planning messages through the Internet and mobile had higher odds of having timely ANC care. - Receiving family planning messages through the Internet increased the odds of antenatal care in urban areas only. - The findings indicate a positive association between media communication regarding family planning and the uptake of maternal healthcare services. | - Due to the cross-sectional nature of the data, the association in the result may not reflect direct causation. - In the result section, it was documented that a very negligible percentage of women received family planning messages through the Internet (3%) and mobile phones (5%). Therefore, the association between the use of mobile phones and the Internet and maternal healthcare utilization may not be feasible. |
| 2 | Which communication technology is effective for promoting reproductive health? Television, radio, and mobile phones in sub-Saharan Africa | F. Iacoella et al.,  2022 | To identify the potential influence of TV or radio ownership as opposed to cell phone ownership on contraceptive use and access to maternal healthcare | - 11 Sub-Saharan African countries - DHS - Cross-sectional at the individual level - 78,000 women (aged 15 to 49 years) - A binary model in the form of a probit regression | - Compared to owning a cell phone, owning a TV or radio has a weaker correlation with better health outcomes. - The study also reveals that access to maternal healthcare is not affected by mobile phone ownership, yet it is a relevant mediator of contraceptive use. - Mobile phones are consistently correlated with better reproductive health behavior. | - The analytical framework of this study removes owners of both assets from the models to compare owners of TV/radio and mobile phones. - Although it is beneficial for the interpretation of results, there is a possibility that the sample may be skewed toward poorer households. |
| 3. | Factors  associated with mobile phone  usage to access maternal  and child healthcare among  women of urban slums in  Dhaka, Bangladesh: a cross-sectional  study | Sabuj Kanti Mistry et al.,  2021 | To explore the  current use of mobile phones for accessing maternal and  child healthcare and its determinants among recently  delivered to women in urban slums of Bangladesh | - Eight slums in Dhaka, Bangladesh - Primary survey - Cross-sectional - 800 recently delivered women - Descriptive analysis and a multilevel binary logistic regression model | - Overall, 73.8% of study participants used mobile phones for accessing maternal and child healthcare. - Participants’ age, husband’s occupation, sex of household head, women’s ownership of mobile phones, and household wealth status were significantly associated with higher odds of using mobile phones to access maternal and child healthcare. - This study reports that mobile phone usage for accessing RMNCH was significantly higher among women who belonged to female-headed households. | - The study finding is not generalizable to the entire slum population as the study was carried out only in one isolated catchment area. - The data were cross-sectional; temporal relationships between the explanatory variables and mobile usage for maternal and child healthcare should be interpreted with caution. - The study findings may be subject to self-reporting and recall biases. |
| 4 | Does women’s mobile phone ownership  matter for health? Evidence from  15 countries | Amnesty E LeFevre et al., 2020 | To assess household and women’s access to phones and  its effects on reproductive, maternal, newborn, and child health (RMNCH) health outcomes | - 15 countries for which data on phone ownership are available - DHS dataset - Cross-sectional at the individual level - 242619 women and 91937 men - Multivariate logistic regression model | - The gender gap in phone ownership is more significant for rural and poorer women. - Women who owned a mobile phone had higher odds of improved reproductive and maternal healthcare, including demand for family planning, 4+ ANC visits, skilled attendance at birth, and tetanus immunization during pregnancy and postnatal care. - Among child health services, phone ownership among women was similarly associated with higher odds of postnatal care for newborns, vitamin A supplementation, and full immunization. | - Methods of adjusting several survey rounds of different countries and other socio-economic backgrounds are not clearly mentioned in the study. - This study also suffers from the negative side of cross-sectional design, i.e., casual inferences may not always be appropriate. |
| 5 | Disparities in mobile phone access and maternal health service utilization in Nigeria: A population-based survey | Larissa Jenningsa, et al., 2015 | To examine if women with limited mobile phone access have differential odds of maternal knowledge and health service utilization compared to female mobile phone users who are currently eligible to participate in maternal mHealth programs | - Nigeria - PATHS2 household survey - Individual-level analysis from the household survey - 3,390 women aged 15–49 years, - Multivariate logistic regression model | - Findings demonstrated that mHealth interventions may not reach women with the lowest levels of maternal awareness and care-seeking in settings with unequal access to mobile phones because these women frequently lacked mobile connectivity. - Women without mobile phone access had significantly lower odds of antenatal care utilization, skilled delivery, and modern contraceptive use compared to mobile phone users. - No differences were observed by mobile phone strata in the uptake of emergency obstetric care, postnatal services, or breastfeeding. | - The study findings are based on a cross-sectional survey design. Therefore, the study could not investigate the causal relationship between mobile phone status and maternal care-seeking and knowledge. - Differences in outcomes may reflect the interventional impact of pre-existing mHealth initiatives, exposure to which was unmeasured in this analysis. |
| 6 | Using mobile phones to promote maternal and child health: knowledge  and attitudes of primary health care providers in southwest Nigeria | Mobolalne Balogun et al.,  2020 | To assess the knowledge and attitudes of primary health care providers on the use of mobile technologies such as Internet-enabled smartphones for maternal and child health services | - Seven urban local government areas in Lagos, Nigeria. - Primary survey - Cross-sectional design - 518 PHC workers (comprising doctors, nurses, midwives, community health officers, and community health extension workers). - Factor analysis, descriptive and multivariate linear and logistic analysis. | - The study showed that having an Internet-enabled smartphone strongly predicted higher knowledge of mHealth among primary healthcare providers. - Primary healthcare providers with higher scores on mHealth knowledge were 1.32 times more likely to use mHealth for maternal and child health services. - Similarly, those with positive attitudes towards mHealth were 1.15 times more likely to use mobile technology for maternal and child health services. | - This study suffers from a cross-sectional design of establishing causal effects. - With this sample design, the study result may not be generalized to the entire healthcare profession. |
| 7 | Community-based maternal, newborn, and  child health surveillance: perceptions and  attitudes of local stakeholders towards  using a mobile phone by village health  volunteers in the Kenge Health Zone,  Democratic Republic of Congo | Mulamba Diese et al.,  2018 | To determine the  perceptions of households, attitudes of community health volunteers, and opinions of nurses in Health centers and  administrative authorities towards the use of mobile phones for MNCH surveillance in the rural KHZ in the DRC | - Democratic Republic of Congo - Primary survey - Mixed method approach (phenomenological and descriptive cross-sectional approaches) - 190 structured interviews and 19 FGD - Thematic analysis and descriptive analysis | - Mobile phones were perceived as quick services for people who needed help; the community’s attitudes towards mobile phone use for data collection, analysis, and use activities were good. - Although some of the community members did not see a direct linkage between this surveillance approach and health benefits, a majority believed that there would be better MNCH services with mobile phone use. - All nurses (participants) estimated that the VHV were willing to participate in the MNCH surveillance using mobile phones and could perform competently if trained well. | - Suffers from subjective bias, thereby posing a reliability threat to the discussion findings. - It was not easy to determine to what extent the interview on the use of mobile phones for MNCH surveillance could have influenced the responses from the interviewees. |
| 8 | Does mobile phone ownership predict  better utilization of maternal and newborn  health services? a cross-sectional study in  Timor-Leste | Juan Nie et al.,  2016 | To investigate socioeconomic factors  and the utilization of maternal and newborn health services  among a population of postpartum women with and  without access to mobile phones in rural Timor-Leste | - Timor-Leste - Baseline survey of Liga Inan mobile phone project of Health Alliance International (HAI) - Individual level analysis (secondary) - 581 women aged 15–49 years with a child under the age of two years - Logistic and multivariate regression models | - Women with mobile phones were significantly more likely to be of higher socioeconomic status and utilize maternal and newborn health services. - However, after adjusting socio-economic factors, household mobile phone ownership was not independently associated with any outcome variables, i.e., use of MNCH services. - The power of mobile phones to help facilitate skilled birth attendance and health facility delivery may not be adequate to overcome greater barriers such as inadequate emergency obstetric transport, long distances from a facility, and other factors such as cultural or social influences. | - The survey on which the study is based did not involve questions related to the purposes of mobile phone usage, which makes it hard to explore whether these women had used their mobile phones to contact health providers, transport, or other women with experience of ANC. - In addition, interviewers did not check health care records to verify the reported care, so there may be over-reporting and recall biases. |
| 9 | Patients’ and Doctors’ Perceptions of a Mobile Phone–Based  Consultation Service for Maternal, Neonatal, and Infant Health  Care in Bangladesh: A Mixed-Methods Study | Mafruha Alam et al.,  2019 | To investigate the ‘Aponjon’ service to understand access, acceptability, usability, benefits, and challenges of a  mobile phone-based consultation service | - Bangladesh - Primary study - Mixed method (quantitative and qualitative) - 3894 unique subscribers of the ‘Aponjon’ service and 11 doctors and 16 subscribers for qualitative purposes. - Descriptive data analysis method and thematic analysis. | - Callers found the consultation service trustworthy, cost-effective, and convenient. - The message service helped families understand the severity of sicknesses and advised them to seek care at health facilities for semi-urgent or urgent conditions. - All callers were satisfied with the consultation service because they could access a trained doctor any time they wanted. - Aponjon doctors identified the lack of connection between the consultation service and available care and emergency services as a key weakness in the service. | - The sample size for interviews was small, which may impact the generalizability of the findings for the whole of Bangladesh. - This research does not address sustainability issues such as operational costs, technological challenges, and revenue generation by the call center. This is vital information for understanding the possibilities of service expansion and escalation. |
| 10 | Women’s Perceptions of Using Mobile Phones for Maternal and  Child Health Support in Afghanistan: Cross-Sectional Survey | Fazal Yamin et al.,  2018 | To determine Afghan women's perceptions regarding using mobile phones for maternal  and child health services | - Nangarhar Province, Afghanistan - Primary survey - cross-sectional level individual-level analysis - 242 participants - Binary and multiple logistic regression | - Most of the participants were willing to receive health messages via a mobile phone. - Automated voice call was the most preferred method for sending health messages. - More than 90% of the women reported that they would like to receive reminders for their children’s vaccinations and antenatal care visits. | - This study was conducted in one province of Afghanistan, which may not represent all women living in the country, especially those in very remote regions or conflict areas. - This study fails to capture the perception of women who have not mobile. - Perceptions of using mobile phones for supporting maternal and child health may differ among women who are new to mobile phone use. |
| 11 | A mobile phone-based multimedia intervention to support maternal health is acceptable and feasible among illiterate pregnant women in Uganda: Qualitative findings from a pilot randomized controlled trial. | Angella Musiimenta et al.,  2021 | To assess the acceptability and feasibility of a mobile phone-based multimedia application (MatHealth app) to support maternal health amongst illiterate pregnant women in rural southwestern Uganda | - Rural southwestern Uganda. - Primary survey of an intervention group - Qualitative analysis - 80 pregnant women initiating antenatal care from Mbarara regional referral hospital were enrolled in a pilot randomized controlled trial and followed until six weeks after delivery. Inductive content analytic approach | - Participants reported that the intervention is acceptable as it enabled them to adopt good maternal health practices, enhanced social support from spouses, provided clinic appointment reminders, and facilitated communication with healthcare providers. - Some participants reported lacking money to cover maternal and child health-related costs such as transport to the clinic, buying the required foods and drinks, purchasing requirements for delivery, and meeting the cost of delivery, especially in case of delivery by operation, which involves extra costs. | - Results may have limited generalizability since they are based on responses of 30 participants who used the MatHealth app and were followed up for six months. - It is not clear how they manifest in larger, diverse contexts and with longer-term follow-up. |
| 12 | Effectiveness of a mobile health intervention  on uptake of recommended postnatal care  services in Nigeria | Aanuoluwapo Omobolanle Olajubu et al.,  2020 | To evaluate the effect of a mobile health  intervention in PNC attendance among mothers in selected primary healthcare facilities in  Osun State, Nigeria | - Osun State, Nigeria - Follow-up survey of an intervention group - quasi-experimental design - Women registered for ANC at the selected PHCs were recruited for the study and were followed up until six weeks after delivery. 175 intervention and 164 control groups. - Descriptive statistics and logistic regression models | - About one-third of respondents in the intervention group had four postnatal care visits, while only 3.7% in the control group had four visits. - After controlling for the effect of confounding variables, group membership remained a significant predictor of PNC uptake. - Mobile health intervention significantly improved the utilization of the recommended four postnatal care visits. | - The lack of blinding could have caused some response bias, especially by the respondents in the intervention group. - The study was geographically restricted to six Local Government Areas in Nigeria. Caution needs to be taken regarding inferences to mothers outside the study settings. |
| 13 | The Social Implications of Technology Diffusion: Uncovering  the Unintended Consequences of People’s Health-Related  Mobile Phone Use in Rural India and China | Marco J. Haenssgen and Proochista Ariana,  2017 | To investigate whether, in the absence of specific mHealth interventions,  people make different healthcare decisions if they use mobile phones during an illness | - Rural Rajasthan (India) and Gansu (China) - Original survey data from rural Rajasthan (India) and rural Gansu (China). - Individual-level analysis - 800 adults sample from the general adult population in 2014. - Single and multi-level logistic, Poisson, and negative binomial regression models with cluster-robust standard errors | - Healthcare access to private providers in Rajasthan and to public providers in Gansu was slightly higher among people who reported using a mobile phone for a health-related purpose during their illness. - Among the phone-aided healthcare-seeking activities, the most common tasks in Rajasthan were exchanging advice, calling a medical practitioner for home treatment, and making appointments; in Gansu, they were home calls, conversations about illnesses, and reassuring peers during the course of an illness. | - The nature of the study does not permit the findings to be extrapolated to other contexts. - The interpretation of the results, therefore, has to pertain specifically to the field sites rather than rural low- and middle-income contexts more generally. |
| 14 | The Digital Divide and Seeking Health Information on Smartphones  in Asia: Survey Study of Ten Countries | Wang et al.,  2022 | To provide a comprehensive profile of mobile health information seekers and to examine  the individual- and country-level digital divide in Asia | - 10 Asian countries - Primary survey conducted by researchers - Individual-level as well as country-level analysis - 9086 smartphone users were recruited. - Multilevel linear regression models | - Respondents who were women, parents, employed, of higher social status, and/or from countries with low health expenditures were likelier to use smartphones to seek health information. - In terms of technology-related factors, the frequency of using smartphones to search for health information was significantly and positively associated with the frequency of smartphone use. | - The sample was not representative and only concentrated on urban areas. - Only examined the role of socioeconomic factors and technology perceptions on mobile health information-seeking |
| 15 | Does having a mobile phone matter? Linking  phone access among women to health in  India: An exploratory analysis of the National  Family Health Survey | Diwakar Mohan et al.,  2020 | To assess the gaps in women’s  access to phones, their influencing factors, and their influence on healthcare  utilization | - India - NFHS-4 - Individual-level analysis - 45,231 women with data on phone access - Multilevel logistic models and Blinder-Oaxaca (BO) decomposition are used to decompose the gaps between women with and without phone access in healthcare utilization | - Phone access in urban areas was positively associated with skilled birth   attendance, postnatal care, and use of modern contraceptives negatively associated with early antenatal care.   - Phone access was not associated with improved utilization indicators in rural settings. - Phone access explained significant gaps in modern contraceptives, moderate gaps in postnatal care and early antenatal care, and smaller differences in the use of skilled birth attendance and immunization. | - The first issue to be addressed is causality since the cross-sectional nature of survey data makes it impossible to determine whether phone access preceded health behaviors. - The survey may not capture the concepts of gender norms, decision-making, and autonomy adequately to address the pathway from mobile phone use to health behavior. - The decomposition methods do not consider the hierarchical nature of the data and the effects of group membership on the level of the outcomes between the groups with and without phone access. |
| 16 | “I am not telling. The mobile is telling”: Factors influencing the outcomes of a community health worker mHealth intervention in India. | Onaedo Ilozumba et al.,  2018 | To identify intervention-related and contextual factors that influence the  observed outcomes of Community Health Workers utilized mHealth intervention called MfM | - Jharkhand State in India - Mobile for Mothers (a quasi-experimental study) - Mixed method approach - 740 women from the intervention site (quantitative), 11 group interviews and 47 individual interviews - Descriptive statistics and content analysis | - Mobile health applications are promising interventions for improving the performance of CHWs and the health-seeking behavior of pregnant women. - CHWs reported that utilizing MfM improved their maternal health knowledge and performance. - In a context where women have limited decision-making power and face multiple challenges in access to care, including financial and socio-cultural barriers and challenges related to health system constraints, mHealth is limited in its ability to address these constraints. - In a context where decisions on maternal health-seeking are characterized by unequal power relations in the household, mHealth interventions that target only women may not lead to expected outcomes. | - Qualitative and qualitative data collection occurred concurrently, so while qualitative interviews provide additional explanations for some quantitative results, some points were not explored in the qualitative interviews. - The primary researcher and other research assistants were not local to the research environment. Simple expressions in Hindi and Kortha (local language) can have nuanced meanings that are difficult to capture in English. |
| 17 | Using m-health to improve healthcare delivery in India: A qualitative examination of the perspective of community health workers and beneficiaries | Lakshmi Gopalakrishnan et al.,  2020 | To examine CHW and beneficiaries' perceptions of a new mHealth intervention (Common Application Software [CAS] for CHWs in India | - Bihar and Madhya Pradesh - Primary survey - Qualitative study design - CHWs (n = 32) and beneficiaries (n = 55) - Thematic analysis using Dedoose | - The mHealth intervention was acceptable to the CHWs who felt that CAS improved their status in the communities where they worked. - The divergent views between CHWs and beneficiaries surrounding the use and impact of CAS highlight an underlying mistrust, socio-cultural barriers in engagement, and technological barriers in implementation. - While technology enables CHWs to improve their service delivery, it does not necessarily help overcome social and cultural barriers that impede CHWs-beneficiary interactions to improve knowledge and health behaviors. | - It is also possible that because these interviews were conducted within six months of introducing CAS, the beneficiaries had not sufficiently engaged with CAS and had not seen the AWWs use CAS in front of them. - It does not examine the contextual factors. |
| 18 | Impact of m-health application used by community health volunteers on improving utilization of maternal, new-born and child health care services in a rural area of Uttar Pradesh, India | Shankar Prinja et al.,  2017 | To understand the impact of an m-health intervention delivered through ASHA workers for improving counseling to pregnant women that is likely to generate demand for MNCH services, thereby improving coverage of services | - Kaushambi district, Uttar Pradesh - ReMiND project (An m-health intervention) - A pre- and post-quasi-experimental design - 450 mothers with a child in the age 29 days to 6 months, and 310 women with a child in the age group of 12–23 months - Propensity score method and difference-in-difference analysis (DID) | - Statistically significant increase in coverage of iron-folic acid supplementation, self-reporting complications during pregnancy and after delivery in the intervention area. - The coverage of three or more antenatal care visits, tetanus toxoid vaccination, full antenatal care, and ambulance usage increased in the intervention area; however, the changes were statistically insignificant. | - Since the choice of the two intervention blocks was not random, it could have resulted in selection bias. - Another limitation of the matching method is the limited number of variables used for matching. |
| 19 | A study to assess the feasibility of Text Messaging Service in delivering maternal and child healthcare messages in a rural area of Tamil Nadu, India | Shib Sekhar Datta, Pandiyan Ranganathan, and Krithiga S Sivakumar,  2014 | To evaluate whether mobile Text Messaging Service is a feasible mode of raising knowledge regarding maternal and child health (MCH) and to explore issues related to mobile text messages as a mode of health education | - Vellore district, Tamil Nadu - A community-based intervention study - Mixed-method approach to assess the effect of the intervention - 120 individuals from 120 households - Descriptive statistics and content analysis via free listing and pile sorting. | - Seventy percent of individuals were willing to receive health information via text messages, and most of them believed text messages could effectively spread health messages. - A significant increase in knowledge related to maternal health was observed following text messages. - Factors related to mobile phone use include minimum economic burden, easy availability, portability, and ease of use. - Factors related to mobile text messages as a mode of health message delivery include direct receipt of information, mass reach, the absence of regional language font in many handsets, and illiterate individuals being unable to read messages. | - The sample size is not a representative one. - Limited health education messages (only MCH care messages) and the text messages sent were also limited, being only script in nature and no pictures. - The result and discussion portion should be well written, including a detailed description of the findings. In this paper, it was written in a concise format. |
| 20 | Use of mobile technology by frontline health workers to promote reproductive, maternal, newborn and child health and nutrition: a cluster randomized controlled Trial in Bihar, India | Suzan L Carmichael et al.,  2019 | To evaluate the impact of a novel mHealth tool that was implemented in Bihar, one of India’s poorest and most populous state | - Bihar, India - The ICT-CCS intervention project - Evaluation study of an Intervention group - Evaluation surveys were conducted with 1100 Frontline Health Workers and 3000 beneficiaries. - Bivariate and difference-in-difference analyses across study groups. | - The ICT-CCS intervention was associated with more frequent coordination of AWWs with ASHAs on home visits and greater job confidence among ASHAs. - The intervention increased Frontline Health Workers' (FLW) antenatal home visits during the third trimester. - In the post-implementation period, postnatal home visits during the first week were increased in the intervention vs the control group. - The intervention also increased skin-to-skin care, breastfeeding immediately after delivery, and age-appropriate complementary feeding. | - A limitation of this study is that some information was not available at both evaluation time points (e.g., certain variables collected from FLWs and maternal reports of frequency of home visits for newborns). - Information on actual health outcomes was not available, such as maternal or infant morbidities or mortality. - Supervision was an important aspect of the intervention, but limited information was collected from FLW supervisors. |
| 21 | Effects of an mHealth voice message service (mMitra) on maternal health knowledge and practices of low-income women in India: findings from a pseudo-randomized controlled trial | Nirmala Murthy et al.,  2020 | To assess the impact of a voice-message-based maternal intervention on maternal health knowledge, attitudes, practices, and outcomes over time | - Mumbai, India - mMITRA program affiliated with the Mobile Alliance for Maternal Action (MAMA) - Pseudo-randomized controlled trial - 1516 women in the intervention group and 500 women in the control group at baseline and post-partum. - descriptive analysis, simple and binary logistic regression | - The intervention group performed significantly better than controls on four maternal health practice indicators: receiving the tetanus toxoid injection, consulting a doctor if spotting or bleeding, saving money for delivery expenses, and delivering in a hospital. - The control group performed significantly better than the intervention group on two practice indicators: resting regularly during pregnancy and having at-home deliveries attended by a skilled birth attendant. - Only one knowledge indicator, on seeking medical care during pregnancy, was statistically increased in the intervention group compared to controls. | - This study relied primarily on self-reported data obtained by survey from women, which could be subject to recall bias and even inaccurate or false reporting by women. - The randomization method is a pseudo one. Therefore, the study may not have been as well balanced as a traditional randomized controlled trial. |
| 22 | Harnessing information technology to improve women’s health information: evidence from Pakistan | Rubeena Zakar et al.,  2014 | To evaluate the capacity of the Information and Communication Center (ICC) in improving the level of health-related information by conducting a cross-sectional survey with women who directly or indirectly sought information from the ICC | - Sialkot, Pakistan - Primary survey - Cross-sectional comparative study - 1140 sample - bivariate analysis and Chi-square test of independence | - The women wanted to receive information on various issues, from family planning, antenatal care, and childcare to garbage disposal and prevention of domestic violence. - Overall, the ICC successfully initiated a meaningful “information dialogue” at the community level, where much-needed information was retrieved, negotiated, mediated, and disseminated through intimate and trusted relations. - The ICC tried to bridge the conceptual and cognitive gap between the modern medical system and the indigenous medical systems. | - The short duration of the project was its limitation. - Women in the direct and no contact groups significantly differ regarding their socio-demographic characteristics, so it was difficult to discern accurate results regarding their knowledge about reproductive and general health issues. - The survey was cross-sectional; hence, researchers could not check whether the health information imparted through the ICC was followed. |
| 23 | How do traditional media access and mobile phone use affect maternal healthcare service use in Bangladesh? Moderated mediation effects of socioeconomic factors | Md Ruhul Kabir,  2022 | To investigate how women’s media and mobile access affect maternal health service (MHS) utilization in Bangladesh | - Bangladesh - Latest DHS - Individual-level analysis - 5,011 ever-married women - Hierarchical logistic regression and moderated mediation analysis are performed to determine the association. | - Only 26.9% of women used mobile for health service use, while more than 55% had media access. - Mobile usage also has a significant association with antenatal and delivery care. - When women have both access to media and mobile, the likelihood of delivering in a health facility increased. - Women’s education mediates the relationship between media and MHS. | - The main limitation of this study is the cross-sectional nature of the data, which limits the ability to determine the causal relationship between variables and their direction. - Self-reported data is another limitation that can introduce respondent and recall bias (under-reporting or over-reporting). |
| 24 | Improving the Quality of Antenatal Care Using Mobile Health in Madagascar: Five-Year Cross-Sectional Study | Anne Caroline Benski et al.,  2020 | To use mHealth to provide antenatal care (ANC) to 1446 pregnant women in a rural area in Madagascar and evaluate the quality of ANC provided by a mHealth system designed to change the behaviors of providers and patients | - Rural Madagascar - Five-Year Cross-Sectional Study - Cross-sectional, observational study - 1446 pregnant women - Descriptive statistics and parsimonious multivariable mixed linear regression model | - After implementing the m-health system, women started to come earlier for their first ANC visit; more women attended their first ANC visit in the second trimester of pregnancy in 2019. - A statistically significant increase in the number of ANC visits per woman from 2015 to 2017; the number of ANC visits per woman became stable after the third year of implementing the PANDA mHealth system. | - The most significant limitation is the lack of a control group to compare with participants using the PANDA mHealth system. - This study did not measure health outcomes to evaluate the effectiveness of the mHealth intervention. |
| 25 | Mobile Phone Messaging to Husbands to Improve Maternal and Child Health Behavior in India | Avishek Hazra, M.E. Khan & Subrato Kumar Mondal,  2018 | To examine whether the provision  of information on maternal and child health to husbands through  their mobile phone would enhance their knowledge and trigger  discussions in their family and to assess whether such discussions would result in the adoption of healthy behaviors | - Jhansi district, Uttar Pradesh - Evaluation survey of an intervention - A quasi-experimental research design with a control group (data collection used a mix of quantitative and qualitative approaches.) - 492 husbands and 10 in-depth interviews (IDIs) of husbands in the experimental area - Univariate descriptive analyses, bivariate and multivariate regression analysis, and thematic analysis using codes. | - Communicating messages using m-Health was an effective intervention for behavior change. - Husbands’ knowledge, controlling for their socio-demographic characteristics, was significantly higher among the listeners of the messages than the non-listeners. - If husbands discussed the messages with family members, the odds of wives practicing health behaviors improved significantly for three behaviors. These include one antenatal check-up in the last trimester of pregnancy, a postnatal check-up within 7 days of delivery, and delayed newborn bathing. | - The intervention was very short duration. A long duration might have a more robust result. - Only by increasing awareness only among one person in the family may not be sufficient. Because this study confirms that only 16% of the husbands (who were interviewed) discussed the messages with their families. |
